# From Prescription to Predicament: A Case of Semaglutide-Induced Discoid Lupus Erythematosus in an Adult Male Patient

**DOI:** 10.7759/cureus.81663

**Published:** 2025-04-03

**Authors:** Kristina Nazzicone, Michael Sidiropoulos, Ashley O'Toole

**Affiliations:** 1 School of Medicine, Queen's University, Kingston, CAN; 2 Department of Dermatology, University of Toronto, Toronto, CAN; 3 Department of Dermatology and Pathology, Odette Cancer Centre, Sunnybrook Hospital, Toronto, CAN; 4 Department of Dermatology, SKiN Centre for Dermatology, Peterborough, CAN; 5 Division of Dermatology, Queen’s University, Kingston, CAN; 6 Department of Dermatology, Probity Medical Research, Waterloo, CAN

**Keywords:** case report, cutaneous lupus erythematosus, discoid lupus erythematosus (dle), glp-1 receptor agonist, semaglutide

## Abstract

We report the case of a 30-year-old male patient who presented with a pruritic, irregular, 4x3 cm scaly purple-red plaque with surrounding papules on his lateral face, scalp, and chin after the introduction of semaglutide (Ozempic). Discoid lupus erythematosus (DLE) was suspected. A punch biopsy of a scalp lesion showed interface changes with loss of pilosebaceous units and follicular plugging, findings consistent with DLE. A complete blood count, antinuclear antibody (ANA), anti-double stranded DNA (dsDNA), and extractable nuclear antigen (ENA) panel was quantified along with erythrocyte sedimentation rate (ESR), C-reactive protein (CRP), complement levels, creatinine, and glomerular filtration rate, ruling out systemic lupus erythematosus (SLE). The patient's condition improved with drug discontinuation as well as topical treatment, including tacrolimus 0.1% ointment and clobetasol lotion. Hydroxychloroquine 200 mg daily was trialed but discontinued due to the patient's concerns about ocular side effects. Follow-up after four months showed improvement, with less scale and erythema. Although semaglutide has been widely used for glycemic control and weight loss, cutaneous adverse effects are seldom reported. This case highlights the potential for drug-induced cutaneous lupus erythematosus (DICLE) associated with immune-modulating medications such as semaglutide, a glucagon-like peptide-1 receptor agonist (GLP-1 RA).

## Introduction

Discoid lupus erythematosus (DLE) is the most common form of chronic cutaneous lupus erythematosus (CCLE), typically affecting women of childbearing age [1,2]. Discoid lupus erythematosus usually presents in sun-exposed areas such as the face, ears, and scalp and poses a high risk of morbidity due to its potential for skin atrophy and scarring [1,3]. The pathophysiology of drug-induced lupus erythematosus (DILE) is incompletely understood yet thought to involve a preexposing genetic or epigenetic susceptibility, instigated as a result of drug biotransformation [4]. Discoid lupus erythematosus is often associated with systemic lupus erythematosus (SLE), as up to 20% of patients with DLE may develop systemic involvement over time. Certain medications, including proton pump inhibitors, hydralazine, and procainamide, have been reported to trigger cutaneous and systemic lupus erythematosus, although no reports of semaglutide-induced DLE have been reported to date [5]. Glucagon-like peptide-1 receptor agonists (GLP-1 RAs), such as semaglutide, are increasingly used for the treatment of type 2 diabetes and obesity, providing metabolic and cardiovascular benefits [6]. The immunomodulating properties of these compounds have also been investigated for their potential to treat or potentiate autoimmune disease [7,8]. Here, we present a case of possible DLE following the initiation of semaglutide (Ozempic) in an adult male patient.

## Case presentation

**Figure 1:**
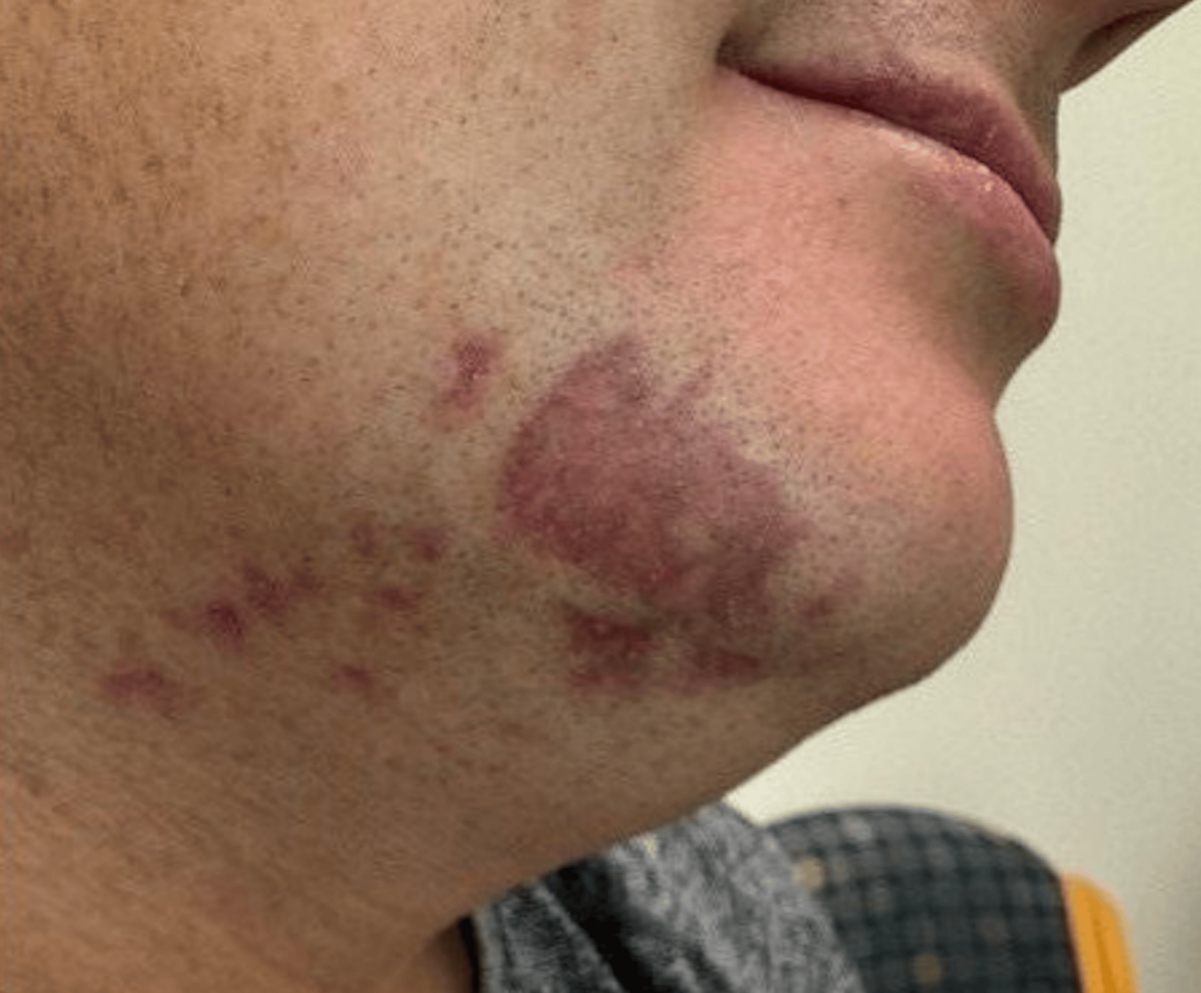
Initial presentation of an irregular, 4x3 cm, scaly, thin, purple-red plaque with surrounding papules on the right chin and upper neck.

A 30-year-old male patient presented to primary care regarding an irregular 4×3 cm scaly and erythematous plaque with surrounding papules on the chin and associated pruritus (Figure 1). The patient first noticed the eruption a few months prior, approximately one week after the initiation of semaglutide (Ozempic) for weight loss. After its initial onset, the lesion grew in size and severity for approximately three weeks before reaching its final presentation. Mupirocin ointment and metronidazole were trialed by the family physician prior to referral, with metronidazole showing moderate success in reducing erythema. The referral stated that fungal scraping was negative, and no other cutaneous or systemic signs or symptoms were reported. Apart from obesity, the patient’s medical history was unremarkable; however, both parents had a history of inflammatory bowel disease. The patient discontinued the semaglutide.

When seen by dermatology, examination revealed erythematous, scaly papules and plaques on the chin, peri-auricular area, and scalp, raising suspicion for localized DLE. The lack of response to mupirocin and metronidazole made infectious causes less likely, prompting further evaluation for autoimmune or drug-induced etiologies. A punch biopsy of the left scalp lesion was performed, and a complete blood count was requested along with antibody titers of double-stranded DNA (dsDNA), antinuclear antibodies (ANA), and an extractable nuclear antigen (ENA) panel. Erythrocyte sedimentation rate (ESR), C-reactive protein (CRP), complement levels, alanine transaminase (ALT), alkaline phosphatase (ALP), creatinine, and glomerular filtration rate were also quantified to assess for systemic disease (Table 1). The patient was prescribed topical tacrolimus 0.1% twice daily to the face and topical clobetasol lotion to the scalp and ears while awaiting results.

**Table 1:** An overview of the patient's laboratory investigation findings. Abnormal value is indicated in bold. The extractable nuclear antigen (ENA) panel includes anti-SS-A, anti-SS-B, anti-RNP, anti-Sm, anti-Scl-70, anti-Jo-1.

Parameters	Patient values	Reference range
White blood cells (WBC)	6.5 × 10⁹/L	4.0 – 11.0 × 10⁹/L
Red blood cells (RBC)	5.24 × 10¹²/L	4.50 – 6.00 × 10¹²/L
Hemoglobin (HGB)	159 g/L	135 – 175 g/L
Hematocrit (HCT)	0.459 L/L	0.400 – 0.500 L/L
Mean corpuscular volume (MCV)	88 fL	80 – 100 fL
Mean corpuscular hemoglobin (MCH)	30.3 pg	27.5 – 33.0 pg
Mean corpuscular hemoglobin concentration (MCHC)	346 g/L	305 – 360 g/L
Red cell distribution width (RDW)	12.3 %	11.5 – 14.5 %
Platelet count	302 × 10⁹/L	150 – 400 × 10⁹/L
Neutrophils	3.3 × 10⁹/L	2.0 – 7.5 × 10⁹/L
Lymphocytes	2.4 × 10⁹/L	1.0 – 3.5 × 10⁹/L
Monocytes	0.6 × 10⁹/L	0.2 – 1.0 × 10⁹/L
Eosinophils	0.2 × 10⁹/L	0.0 – 0.5 × 10⁹/L
Basophils	0.0 × 10⁹/L	0.0 – 0.2 × 10⁹/L
Immature granulocytes	0.0 × 10⁹/L	0.0 – 0.1 × 10⁹/L
Nucleated red blood cells (NRBC)	0/100 WBC	0/100 WBC
Double-stranded DNA (dsDNA)	<1 IU/mL	<5 IU/mL
Antinuclear antibody (ANA)	Negative	Negative at 1:80 titre
Extractable nuclear antigen (ENA) panel	Negative	Positive at any quantity
Erythrocyte sedimentation rate (ESR)	2 mm/hr	2-30 mm/hr
C-reactive protein (CRP)	3.3 mg/L	<5.0 mg/L
Complement C3	1.77 g/L	0.9-1.8 g/L
Complement C4	0.3 g/L	0.15-0.53 g/L
Creatinine	82 umol/L	67-117 umol/L
Estimated glomerular filtration rate (eGFR)	110 mL/min/1.73m2	>90 mL/min/1.73m2
Alkaline phosphatase (ALP)	103 U/L	40-129 U/L
Alanine transaminase (ALT)	99 U/L	<50 U/L

**Figure 2:**
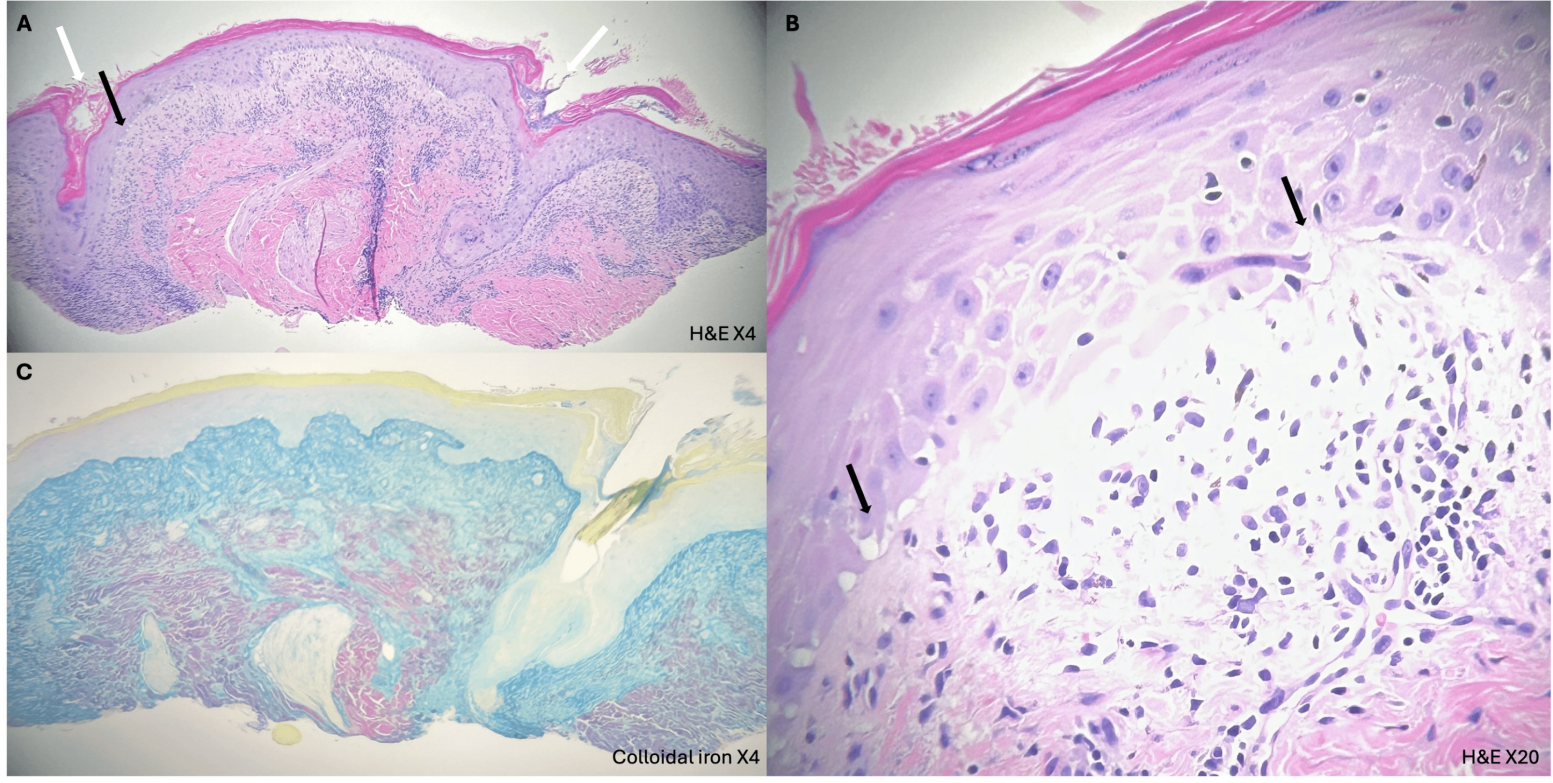
Histopathology of left scalp biopsy (A) Hematoxylin and eosin (H&E) stained section (X4) showing vacuolar interface inflammation (black arrows), follicular plugging (white arrows), and perifollicular and periadnexal inflammation; (B) H&E-stained section (X20) showing similar vacuolar interface inflammation (black arrows); (C) Colloidal iron stain (X4) showing significant mucin deposits. Significant loss of pilosebaceous units is also noted.

**Figure 3:**
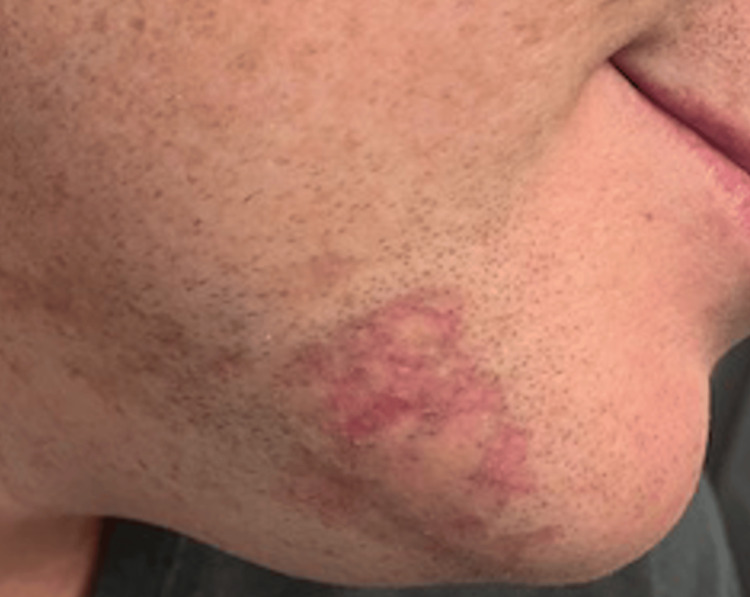
Right chin and neck lesions four months after the initiation of topical tacrolimus 0.1% and clobetasol lotion; scale and erythema are improved, but residual hyperpigmentation and erythema can be appreciated.

Pathology demonstrated an interface process with a superficial lymphoid infiltrate and basal cell layer vacuolar degeneration with necrotic keratinocytes. A periadnexal and perivascular infiltrate with significant loss of pilo-sebaceous units and follicular plugging was also noted (Figures 2A, 2B). A colloidal iron stain showed dermal mucin (Figure 2C). Basement membrane thickening was demonstrated by periodic acid-Schiff (PAS) and PAS-diastase (PAS-D) stains. All the above-stated bloodwork was within normal limits apart from slightly elevated ALT at 99 U/L. The remainder of the investigations were negative, ruling out comorbid SLE. Hydroxychloroquine (Plaquenil) 200mg was initiated along with the continuation of topical medications. At the four-month follow-up, hydroxychloroquine was discontinued due to patient concerns about ocular side effects, and clobetasol was mildly successful at relieving pruritus. The skin was improved with less scale and erythema (Figure 3). The patient remains off the semaglutide yet continues to have residual scarring and post-inflammatory hyperpigmentation.

## Discussion

Drug-induced cutaneous lupus erythematosus (DICLE) is a cutaneous lupus variant occurring days to years following exposure to a culprit drug in those without a history of autoimmune disease [4,9]. A diagnosis of DICLE is usually made according to the clinical plausibility of the temporal relationship between the eruption and initiation of a new medication, physical examination, serology, and lesion biopsy [4]. Histopathology of discoid lesions demonstrates periadnexal inflammation, hyperkeratosis, follicular plugging, and interface dermatitis (i.e., basal cell damage). Dermal mucin deposits and basement membrane thickening can also be appreciated [10]. Discontinuation of the culprit medication should be the first step in the management of all forms of DILE, as manifestations usually subside following the removal of the offending agent. Patients presenting with DICLE should also be evaluated for SLE at diagnosis and in the ensuing weeks to months [4].

Semaglutide is a GLP-1 RA widely used for the management of type 2 diabetes and weight loss. These incretin mimetics function to augment the actions of insulin, inhibit those of glucagon, and decrease gastric emptying, mimicking the actions of endogenous GLP-1 released from gut neuroendocrine cells [6]. The most commonly reported side effects of GLP-1 RAs are gastrointestinal, with 10% to 50% of patients experiencing nausea, vomiting, and diarrhea. Given the recent surge in popularity of this compound, its potential cutaneous adverse events are still being elucidated [11].

Emerging evidence suggests GLP-1 RAs might act to dampen the effects of the immune system through pathways independent from those promoting weight loss and glycemic control. Recent retrospective data have shown a modest but statistically significant reduction in the incidence of systemic inflammatory diseases, including SLE, among those using GLP-1 RAs [12]. GLP-1 RAs have been shown to inhibit the secretion of IFN-gamma and IL-4 by invariant natural killer T (iNKT) cells, which are key mediators between the innate and adaptive immune systems. These cytokine alterations have primarily been linked to psoriasis and have demonstrated dose-dependent benefits on disease severity [13].

A recent study found a positive association between genetically proxied GLP-1 receptor activation and an increased risk of developing SLE [7]. GLP-1 RAs have been shown to increase double-negative B cells, a subpopulation of B cells frequently implicated in autoimmune diseases, including SLE. These findings might provide a plausible mechanism to support how GLP-1 RAs may contribute to an increased risk of developing SLE or exacerbate preexisting autoimmune disease [7].

The pathophysiology of DICLE is not fully understood but is thought to involve genetic, epigenetic, and pharmacokinetic involvement [4]. Similar to idiopathic CCLE, both human leukocyte antigen (HLA) variants and alterations to key players in the complement cascade have been suggested as plausible mechanisms; however, findings across studies are not always consistent nor meant to represent definitive consensus [4,8]. Over 40 medications have been reported as probable culprits for DILE, several of which belong to a class of immunomodulating medications [14].

A case of DILE caused by semaglutide (Wegovy) was published less than a year ago [15]. The patient presented with diffuse petechial rash in the lower extremities and abdomen but did not appear to show signs of DLE. As of March 15, 2025, 60 prescriber and user reports of skin and subcutaneous tissue adverse events occurring in patients using semaglutide have been submitted to Health Canada [16]. This number is much higher at 3,271 events in the United States, according to Food and Drug Agency (FDA) data [17]. Unsurprisingly, most of these reports were filed in the last decade. Neither database reported on semaglutide-induced DLE explicitly; however, descriptions of skin atrophy and pigmentation changes can be appreciated, and a single case of “lupus-like syndrome” was reported to the FDA in November 2024. It is unclear if this case represents systemic or cutaneous lupus [17].

## Conclusions

To our knowledge, this study reports a potential case of semaglutide-induced DLE in a young adult male patient. Given the proven efficacy of GLP-1 RAs in treating obesity and diabetes and their potential for further therapeutic development, the number of patients who will be prescribed this medication is likely to increase. Further research and post-market surveillance, including dermatologic monitoring, are needed to better characterize the mechanism of semaglutide-induced DLE.
